# Converging Inflammations: Simultaneous Myositis and Polyneuropathy as a Diagnostic Challenge

**DOI:** 10.7759/cureus.79536

**Published:** 2025-02-23

**Authors:** Shikha Kumari, Gudimetla Priyanka, Kiran N C, Rachit Bansal, Siddharth Sharma

**Affiliations:** 1 General Medicine, Vardhman Mahavir Medical College and Safdarjung Hospital, New Delhi, IND

**Keywords:** antisynthetase syndrome, inflammatory myositis, neuromyositis, polymyositis, polyneuropathy

## Abstract

Idiopathic inflammatory myopathies consist of a variety of autoimmune diseases with variable clinical manifestations, treatment response, and prognosis. Symmetrical proximal predominant muscle weakness is the usual presenting clinical manifestation except in the case of clinically amyopathic dermatomyositis and inclusion body myositis, which represent without muscle weakness and asymmetric muscle weakness, respectively. Other extramuscular manifestations include skin rash and necrosis, arthritis, interstitial lung disease, myocarditis, dysphagia, and Raynaud’s phenomenon. However, there are only a few case reports of neuromyositis in the literature. Here, we describe a case of a 30-year-old Indian male patient with polymyositis associated with inflammatory polyneuropathy.

## Introduction

Inflammatory myopathies (IMs), or systemic autoimmune myopathies, refer to a diverse range of illnesses that are frequently associated with other organ involvement and symmetrical proximal predominant muscle weakness. The five main types of IMs are dermatomyositis (DM), immune-mediated necrotizing myopathy (IMNM), anti-synthetase syndrome (ASS), anti-mitochondrial M2-associated myopathy, and inclusion body myositis (IBM). Other IMs include those caused by infection, checkpoint inhibitors, and eosinophilic, granulomatous, and metabolic myopathies. Elevated levels of blood muscle enzymes, myopathic electromyograms, and varying degrees of inflammation and regeneration observed in histological inspections are commonly found in myopathies, along with other symptoms. Furthermore, the discovery of various autoantibodies, categorized into myositis-specific antibodies (MSA) and myositis-associated antibodies (MAA), helps establish the diagnosis of myositis. MSA are autoantibodies that are specific to myositis, whereas MAA are autoantibodies that may also be present in other autoimmune disorders, such as Sjögren syndrome, systemic lupus erythematosus (SLE), and scleroderma. Myocarditis, interstitial lung disease, malignancies, and other connective tissue disorders are common illnesses linked to IMs, as are DM, IMNM, and IBM [1,2].

## Case presentation

A 30-year-old Indian male patient without any comorbidities and prior history of any chronic illness presented to the medicine department with complaints of weakness in all four limbs for the past eight months. The weakness was insidious in onset, gradually progressive, and persistent, with symmetrical involvement (proximal more than distal). It was associated with severe asthenia and muscle pain but not associated with diurnal variation, fatigue, or cola-coloured urine. Additionally, the patient reported dyspnea on exertion and a non-productive cough for six months, as well as difficulty in swallowing for one and a half months. There was no history of altered sensorium, seizure, headache, nausea, vomiting, diminished visual acuity, periorbital pain associated with eye movement, diplopia, ptosis, hiccups, sensory levels associated with band-like sensation or tightening around the torso, or any muscle twitching, fasciculation, and spasm. There was also no history suggestive of bladder or bowel involvement, cranial nerve involvement, incoordination or instability, or abnormal body movement.

However, the patient did have a history of experiencing multiple joint pains, not accompanied by swelling, tenderness, morning stiffness, deformity, or contracture. There was no history of skin rash, tightening, dry eyes (xerophthalmia), dry mouth (xerostomia), dental issues, Raynaud’s phenomenon, mechanic's hands, or hiker's feet. Additionally, the patient reported having undergone triple drug therapy (prednisolone, hydroxychloroquine, and methotrexate) for approximately four months, as prescribed by a private practitioner to address the joint pain.

Examination and diagnosis

The patient had an average build, was well-nourished, and presented with myoedema without signs of pallor, jaundice, cyanosis, clubbing, lymphadenopathy, wasting, or any neurocutaneous markers. The sensorium was intact. The patient was right-handed and showed no abnormalities in speech. During the motor examination, generalized hypotonia was noted. According to the Medical Research Council (MRC) grading system, muscle power was rated as follows: shoulder shrug - 3/5, all joints in the upper limbs - 3/5, and all joints in the lower limbs - 2/5. Additionally, a head drop was noted. All superficial reflexes were intact; bilateral plantar were flexor in response. Deep tendon reflexes (DTR) were absent in the upper limbs, with hyporeflexic ankles and knee jerks. All sensory modalities were intact, with no signs of meningeal irritation or cerebellar abnormalities. There was no deformity of the skull or spine. The respiratory system examination revealed bilateral infra-axillary velcro crepitations. No organomegaly was detected, and the cardiovascular system was within normal limits.

Laboratory investigations revealed an elevated erythrocyte sedimentation rate (ESR) of 79 mm/hr and a C-reactive protein (CRP) level of 60 mg/dl. Liver enzymes were elevated, with aspartate transaminase (AST) at 330 U/L, alanine aminotransferase (ALT) at 259 U/L, and alkaline phosphatase (ALP) at 80 U/L. The total protein was 5.4 g/dl, serum albumin was 2.0 g/dl, and creatine kinase was significantly elevated at 3473 U/L. The complete blood count, absolute eosinophil count, renal function tests, serum electrolytes, thyroid profile, serum vitamin D levels, and serum parathyroid hormone (PTH) levels were all within the normal range. Viral markers for HIV, hepatitis B surface antigen (HBsAg), and anti-hepatitis C virus (anti-HCV) antibodies were non-reactive, while the purified protein derivative (PPD) test was negative. Cerebrospinal fluid (CSF) analysis revealed glucose at 61 mg/dl and protein at 41 mg/dl, with no cells seen on cytology. Rheumatoid factor, anti-cyclic citrullinated peptide (anti-CCP) antibodies, and anti-nuclear antibodies (ANA), as tested by both enzyme-linked immunosorbent assay (ELISA) and indirect immunofluorescence assay (IFA), were negative. The anti-neutrophil cytoplasmic antibodies (ANCA) profile was also negative. The extractable nuclear antigen (ENA) profile (line immunoassay) was positive for Ro-52 and strongly positive for Jo-1. The myositis profile showed strong positivity for anti-Jo-1 and histidyl t-RNA synthetase, with a weak positive result for anti-Ro-52. The autoimmune neuropathy antibodies profile was not performed due to logistic issues (Table 1).

**Table 1:** Laboratory reports ALT, alanine transaminase; ALP, alkaline phosphatase; ANA, anti-nuclear antibodies; ANCA, anti-neutrophil cytoplasmic autoantibody; Anti-CCP, anti-cyclic citrullinated peptide; Ab, antibodies; AST, aspartate transaminase; CRP, C-reactive protein; CSF, cerebrospinal fluid; ESR, erythrocyte sedimentation rate; HBsAg, hepatitis B surface antigen; HCV, hepatitis C virus; PPD, purified protein derivative; ELISA, enzyme-linked immunosorbent assay; IFA, indirect immunofluorescence assay

Parameter	Values	Normal Range
ESR (mm/hr)	79	0-20
CRP (mg/dL)	60	
AST (U/L)	330	0-40
ALT (U/L)	259	0-40
ALP (U/L)	80	40-140
Serum albumin (g/dL)	2	3.5-5.0
Total protein (g/dl)	5.4	6.4-8.3
Serum creatine kinase (U/L)	3473	26-192
Serum urea (mg/dL)	20	17-43
HBsAg and anti-HCV Ab	Non-reactive	
PPD test	Negative	
Rheumatoid factor	Negative	
Anti-CCP Ab	8	<20
CSF glucose (mg/dL)	61	50-80
CSF protein (mg/dL)	41	15-45
ANA (ELISA and IFA)	Negative	
ANCA profile	Negative	
dsDNA	Negative	
Ro-52	Positive	
Jo-1	Positive	
Myositis profile		
Anti-Jo-1 and histidyl t-RNA synthetase	Strongly positive	

Venous color Doppler ultrasonography of both lower limbs showed no evidence of deep vein thrombosis (DVT). Two-dimensional echocardiography was normal. A nerve conduction study suggested pure motor mixed (axonal plus demyelinating) polyneuropathy (Tables 2-4).

**Table 2:** Motor nerve conduction study ADM, abductor digiti minimi; AH; abductor hallucis; APB, abductor pollicis brevis; EDB, extensor digitorum brevis; Fib, fibula; L, left; NR, non-reactive; Pop, popliteal; R, right

Motor Nerve Conduction Study								
Nerve/site	Recording site	Latency (ms)	Amplitude (mV)	Segments	Distances (mm)	Latency difference (ms)	Velocity (m/s)	Duration (ms)
R Median-APB Wrist	APB	3.59	3.7	Wrist-APB				10.63
R Median-APB Elbow	APB	7.86	3.5	Elbow-Wrist	210	4.27	49	10.78
L Median-APB Wrist	APB	3.39	3.2	Wrist-APB				10.78
L Median-APB Elbow	APB	8.13	3.2	Elbow-Wrist	220	4.74	46	10.36
R Ulnar-ADM Wrist	ADM	2.34	3.3	Wrist-ADM				5.89
R Ulnar-ADM Elbow	ADM	6.82	3.3	Elbow-Wrist	250	4.48	56	5.57
L Ulnar-ADM Wrist	ADM	2.5	3.3	Wrist-ADM				8.18
L Ulnar-ADM Elbow	ADM	6.25	3.1	Elbow-Wrist	250	3.75	67	7.97
R Peroneal-EDB Ankle	EDB	NR	NR	Ankle-EDB				NR
R Peroneal-EDB Fib Head	EDB	NR	NR	Fib Head-Ankle	NR	NR		NR
L Peroneal-EDB Ankle	EDB	NR	NR	Ankle-EDB				NR
L Peroneal-EDB Fib Head	EDB	NR	NR	Fib Head-Ankle	NR	NR		NR
R Tibia-AH Ankle	AH	3.96	3.8	Ankle-AH				6.72
R Tibia-AH Pop Fossa	AH	12.92	3.3	Pop Fossa-Ankle	370	8.96	41	6.82
L Tibia-AH Ankle	AH	4.06	5.6	Ankle-AH				7.45
L Tibia-AH Pop Fossa	AH	4.79	4.79	Pop Fossa-Ankle	390	0.73	535	12.6

**Table 3:** Sensory nerve conduction study L, left; R, right; Dig, digit

Sensory Nerve Conduction Study							
Nerve/site	Record site	Latency (ms)	Amplitude (µV)	Segments	Distance (mm)	Velocity (m/s)	Duration (ms)
R Median-Digit II (Antidromic)							
Wrist	Digit II	2.71	21.6	Wrist-Dig II	130	48	1.98
L Median-Digit II (Antidromic)							
Wrist	Digit II	2.92	25.9	Wrist-Dig II	130	45	1.77
R Ulnar-Digit V (Antidromic)							
Wrist	Digit V	2.14	21.1	Wrist-Dig V	110	52	1.56
L Ulnar-Digit V (Antidromic)							
Wrist	Digit V	2.08	25.3	Wrist-Dig V	100	48	1.41
R Sural-Ankle (Calf)							
Calf	Ankle	2.55	7.4	Calf-Ankle	120	47	1.56
L Sural-Ankle (Calf)							
Calf	Ankle	2.81	7.3	Calf-Ankle	130	46	1.56

**Table 4:** F-wave ADM, abductor digiti minimi; AH, abductor hallucis, APB, abductor pollicis brevis; L, left; Lat, latency; Max, maximum; Min, minimum; R, right

F-Wave			
Nerve	Min F Lat (ms)	Max F Lat (ms)	Mean F Lat (ms)
R Median-APB	28.02	28.7	28.3
R Ulnar-ADM	24.69	25.05	24.87
L Median-APB	27.5	27.6	27.55
L Ulnar-ADM	26.46	26.77	26.56
R Tibial-AH	44.01	45.16	44.52
L Tibial-AH	42.86	43.49	43.26

Electromyography results revealed short duration and low amplitude motor unit action potential (MUAP) with early recruitment in the proximal muscles; there was no insertional activity (Table 5).

**Table 5:** Electromyography L, left; MUAP, motor unit action potential; N, normal; PSW, positive sharp wave; Poly, polyphasic potential; R, right; Ia, insertional activity

Electromyography Summary								
Muscle	Spontaneous				MUAP			Recruitment
	Ia	Fibrillations	PSW	Fasciculations	Amplitude	Duration	Poly	Pattern
R Deltoid	N	None	None	None	0.3-0.6 mV	<5 ms	Many	Early
R First dorsal interosseous	N	None	None	None	0.5-0.8 mV	<5 ms	Few	Early
R Vastus lateralis	N	None	None	None	0.3-0.6 mV	<5 ms	Few	Early
R Tibialis anterior	N	None	None	None	0.3-0.6 mV	<5 ms	Few	Early

**Figure 1:**
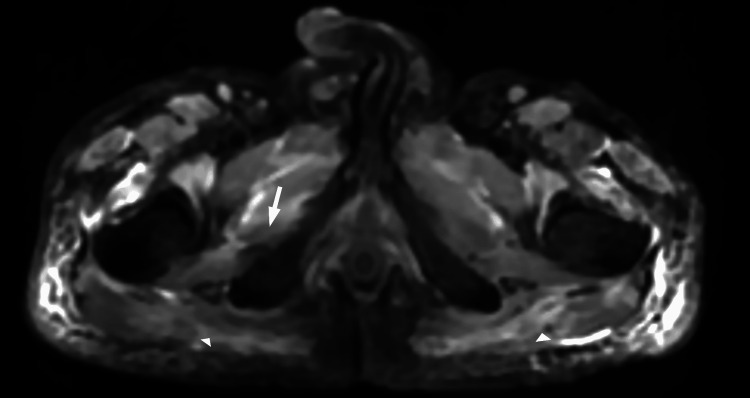
MRI pelvis Inflammation and atrophy of the right obturator externus (arrow) and bilateral gluteus maximus (arrowheads).

The MRI of the shoulder and pelvic girdle myogram exhibited bilateral asymmetric involvement of the upper and lower limb girdle muscle (LL>UL), with edema and inflammatory changes in the muscles and subcutaneous tissue, along with atrophy of the right obturator externus and bilateral gluteus maximus muscles, suggestive of an early chronic stage (Figure 1).

**Figure 2:**
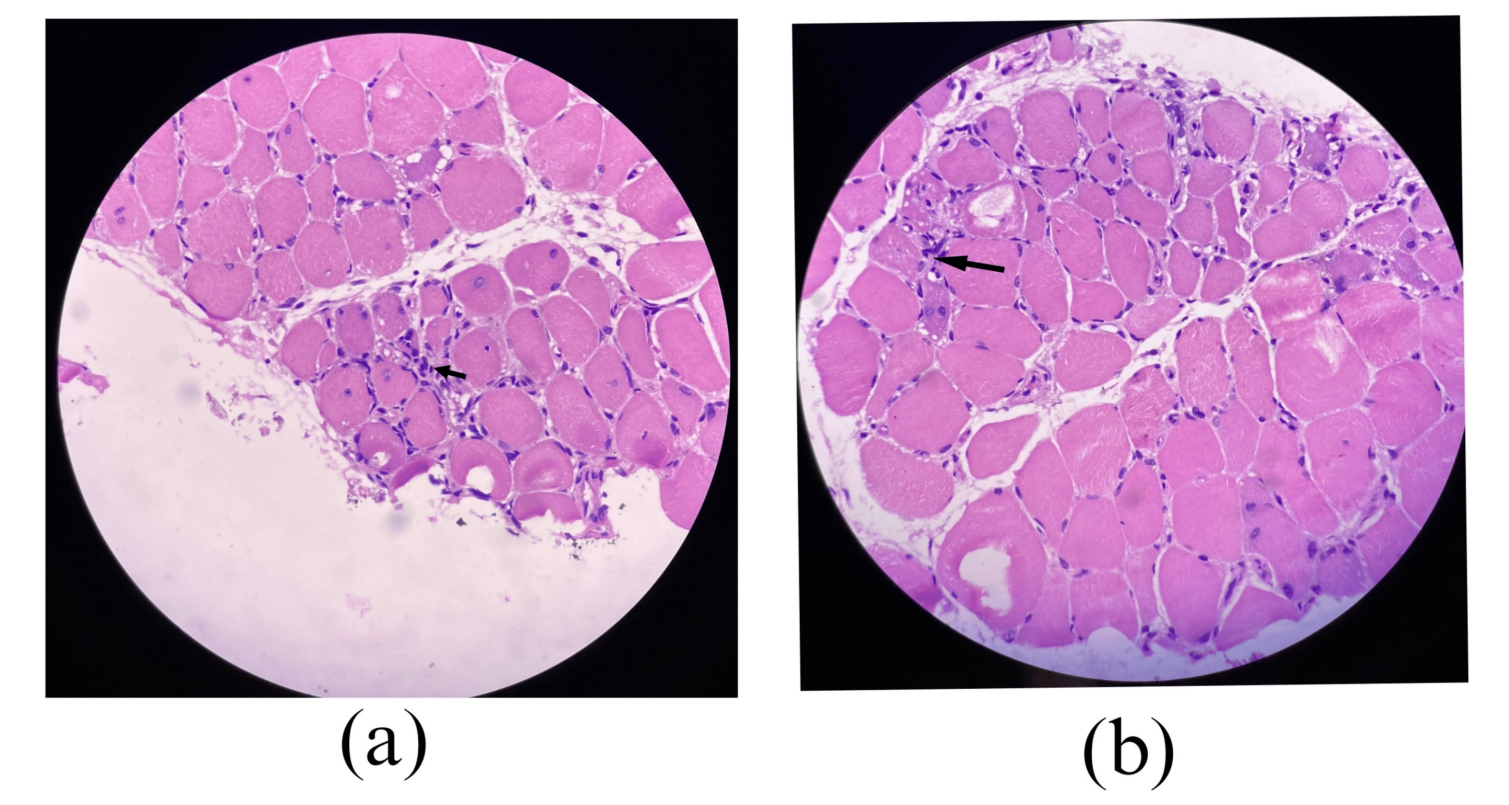
Muscle biopsy of the left calf muscle (a,b) showing endomysial inflammation composed of lymphocytes, plasma cells, and a few macrophages (arrows).

Muscle biopsy (left calf muscle from the gastrocnemius) showed mild variation in muscle fiber size, with a few fibers exhibiting internalized nuclei. The endomysial inflammation consisted of lymphocytes, plasma cells, a few macrophages, and several basophilic regenerating myofibers with basophilic cytoplasm and large nuclei. Necrosis was not observed. These features were consistent with polymyositis (Figures 2a-2b).

Immunohistochemistry of the muscle and nerve biopsy could not be performed due to logistical issues.

**Figure 3:**
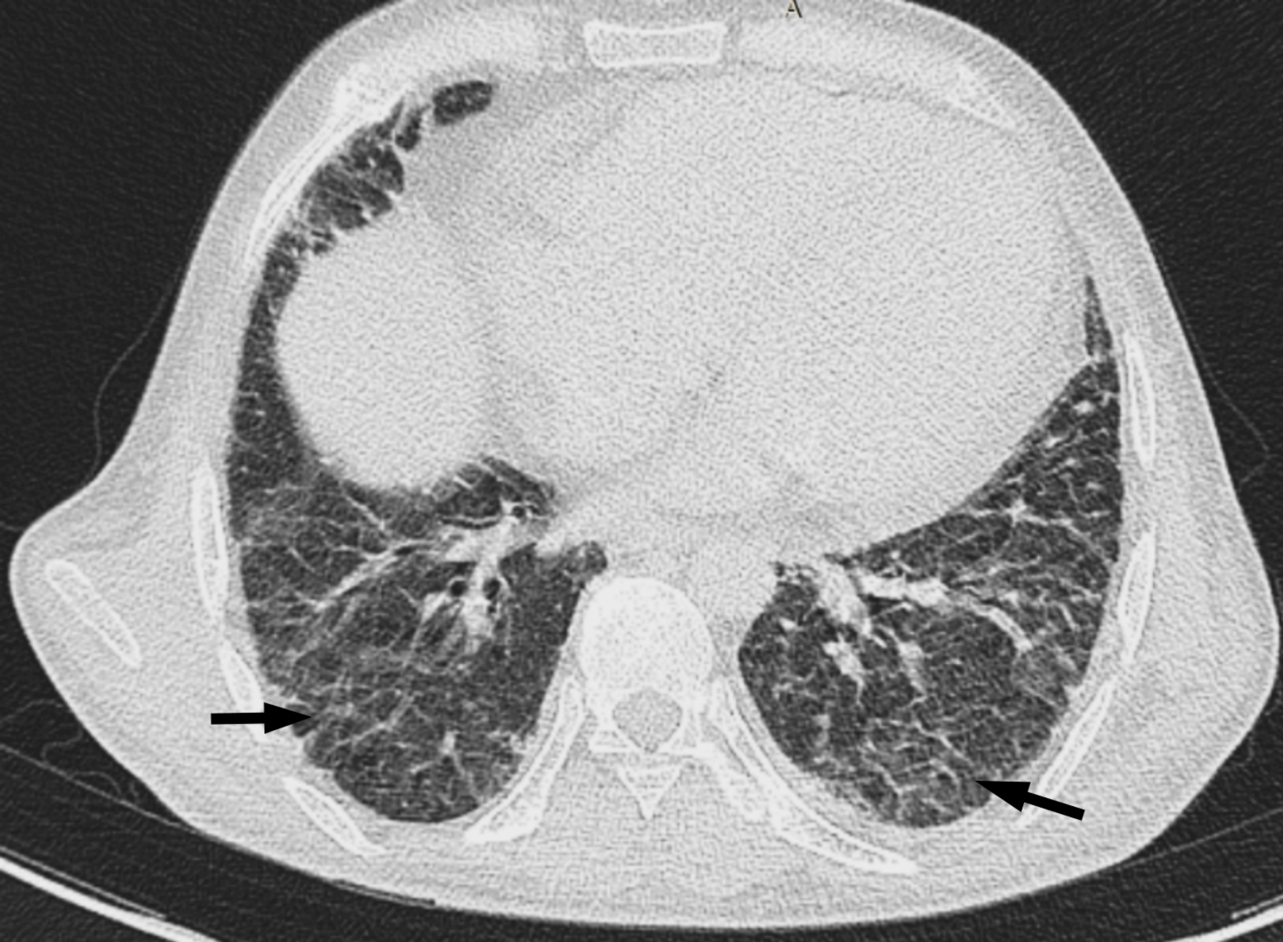
CT chest Bilateral subpleural reticular opacities (arrows).

A CT scan of the chest suggested reticular opacities in the bilateral lung fields, predominantly in the subpleural and peribronchovascular regions, as well as a peri-lobular pattern with opacities in the periphery of the pulmonary lobules in the bilateral lower lobes. The possibilities included interstitial lung disease, likely organizing pneumonia/fibrosis, or nonspecific interstitial pneumonitis (Figure 3).

The patient was diagnosed with overlap syndrome, which involves both inflammatory myositis and inflammatory polyneuropathy. Due to concerns about potential steroid-induced myopathy, corticosteroids were initially withheld; following this decision, the patient's strength improved.

Logistical issues prevented the administration of intravenous immunoglobulin (IVIG) and plasma exchange (PLEX) therapy. Instead, the patient was treated with rituximab according to body surface area (BSA), receiving two doses of 500 mg administered two weeks apart. As a result, his weakness improved significantly; muscle strength was measured at 4/5 in the lower limbs and 4+/5 in the upper limbs. He could also raise his head and hold it in an upright position independently. Additionally, there was an improvement in his transaminase levels, and his CRP test returned negative results. His dyspnea improved from MRC grade 4 to grade 1. The patient was discharged with a recommendation for regular follow-up appointments.

## Discussion

Chronic muscular weakness, muscle exhaustion, and the infiltration of mononuclear cells into skeletal muscle are symptoms of a diverse group of systemic autoimmune rheumatic disorders known as inflammatory muscle diseases or IMs. These diseases primarily affect the muscles of the trunk and proximal limbs, with or without skin involvement. IMs are generally accepted to have autoimmune origins, often associated with other autoimmune diseases such as collagen vascular diseases and Hashimoto's thyroiditis. More than 50% of patients with IMs have specific autoantibodies, some associated with myositis and others merely related to it. These are commonly referred to as MAA and MSA [1,2].

In the literature, various conditions associated with myositis have been well described, including myocarditis, interstitial lung disease, malignancies, and other connective tissue diseases linked with DM and polymyositis. Also, statin use can trigger cases with necrotizing myopathy (NM), while other associations include non-erosive arthritis, interstitial lung disease, Raynaud’s phenomenon, mechanic's hands, and fever with the ankylosing spondylitis (AS), as well as granular lymphocytic leukemia/lymphocytosis, dryness (sicca), or Sjögren’s syndrome with IBM.

Senator was the first to describe the association of myositis with neuritis, coining the term "neuromyositis." Since then, only a few cases have been reported where inflammatory myositis overlaps with inflammatory polyneuropathy [3]. In 2003, Matsui et al. reported two cases with simultaneous diagnoses of DM and inflammatory neuropathy. Polyneuropathy was observed in both patients, and cerebral vasculitis was suspected in one. Histopathological examination of biopsied specimens from skeletal muscle, skin, and sural nerve revealed vasculitis [4]. In 2010, Nomura et al. described a case in which DM and severe axonal neuropathy were diagnosed simultaneously. Histopathological evaluation of a nerve biopsy revealed axonal shrinkage without demyelination or evidence of vasculitis [5]. In 2011, Reimann et al. reported a case in which myositis, neuropathy, pipestem capillaries, and vascular-activated complement deposition were all diagnosed at the same time [6]. In 2016, Mathis et al. reported a case of triple overlap syndrome of myositis, polyneuropathy, and myasthenia gravis [7].

We highlight the complexity of diagnosing neuromuscular disorders that share symptoms with other autoimmune conditions. Recognizing these unusual occurrences is crucial for effectively treating these patients.

## Conclusions

This case highlights the rare simultaneous occurrence of inflammatory myositis and polyneuropathy, emphasizing the complexities of diagnosing neuromyositis. The patient’s clinical presentation and specific laboratory findings support this diagnosis. Early recognition and appropriate management are crucial for improving outcomes in such patients. Further research is needed to understand the underlying mechanisms and to optimize therapeutic strategies for similar cases. A multidisciplinary approach is essential for providing comprehensive care.
